# Homocysteine levels in women with a history of gestational diabetes mellitus

**DOI:** 10.1186/s13098-015-0088-2

**Published:** 2015-10-26

**Authors:** Piotr Molęda, Aneta Fronczyk, Krzysztof Safranow, Lilianna Majkowska

**Affiliations:** Department of Diabetology and Internal Medicine, Pomeranian Medical University in Szczecin, Siedlecka 2 Str, 72-010 Police, Poland; Department of Biochemistry and Medical Chemistry, Pomeranian Medical University in Szczecin, Powstancow Wielkopolskich Av. 72, 71-899 Szczecin, Poland

**Keywords:** Glucose intolerance, Homocysteine, Insulin resistance, Previous gestational diabetes

## Abstract

**Background:**

Previous gestational diabetes (pGDM) is a risk factor of type 2 diabetes, hypertension and cardiovascular diseases. Homocysteine is one of markers of cardiovascular risk. The aim of this study was to assess the homocysteine levels in women with pGDM and to evaluate its relationship with current carbohydrate metabolism and nourishment status.

**Methods:**

The study group comprised 199 women at 7.8 ± 1.0 years after pGDM and 50 control women in whom pGDM was excluded. The analyzed parameters: BMI, WHR, body composition (Tanita SC-330S analyzer), glucose and insulin levels in oral glucose tolerance test (OGTT), insulin resistance index (HOMA-IR), HbA1c, lipid profile, homocysteine, creatinine and creatinine clearance. The Mann–Whitney test and Chi–squared test were used for comparison of continuous and nominal variables, respectively. Correlations between continuous variables in each group were analyzed using Spearman’s rank correlation coefficient (Rs). A logarithmic transformation was applied for variables with non-normal distribution.

**Results:**

There were no differences between the pGDM women and controls in terms of age, number of childbirths, time from indexed pregnancy, pre-pregnancy BMI, or current anthropometric parameters. In pGDM women HbA1c and all glucose levels in OGTT were significantly higher, but still within the normal range. No significant differences were found in homocysteine levels, HOMA-IR, blood lipids, creatinine and creatinine clearance. Homocysteine levels did not differ significantly in subgroups categorized according to the current OGTT results or BMI. Carbohydrate metabolism disorders, overweight and obesity were associated with higher creatinine clearance. Positive correlation between homocysteine and creatinine (r = 0.21, p < 0.004), and a negative correlation with creatinine clearance (r = −0.16, p < 0.03) were found.

**Conclusions:**

In women with pGDM, homocysteine is not a marker of glucose tolerance disturbances and cardiovascular risk. Increased glomerular filtration rate, observed in more severe disorders of carbohydrate metabolism and greater BMI, may temporarily protect against an increase of proatherogenic homocysteine.

## Background

A history of gestational diabetes (GDM) is associated with an increased risk of type 2 diabetes, hypertension and cardiovascular diseases [1–4]. Women with GDM are characterized by increased insulin resistance, which is a risk factor for atherosclerosis [5]. Early atherosclerotic lesions in women with GDM are manifested by thickening of the intima media complex as revealed by ultrasound imaging [6]. The increased levels of proinflammatory cytokines observed in pregnancies complicated by GDM indicate the presence of subclinical inflammation [7].

Homocysteine (Hcy) is one of the markers of increased risk of cardiovascular diseases [8]. The proatherogenic activity of Hcy results from its cytotoxic effect on endothelial cells because it induces or promotes the formation of atherosclerotic plaque [9]. Increased Hcy levels promote oxidative stress, have prothrombotic activity, affect the proliferation of vascular smooth muscle cells, and induce/promote subclinical inflammation [10–12]. It has also been suggested that Hcy contributes to the development of insulin resistance associated with the metabolic syndrome, type 2 diabetes and atherogenesis [13–15].

There is a very limited number of studies on homocysteine levels in women with GDM, but those existing report both normal and increased Hcy levels [6, 16–18]. Even less information can be found on Hcy in women with a history of GDM (pGDM), and these are single reports from studies on populations from Korea and Iran that indicated increased homocysteine levels in women several years after diagnosis of pGDM that correlated with an increased risk of developing type 2 diabetes [19, 20]. To date, no reports have been available on Hcy in women with pGDM in a European population. Moreover, no information is available on Hcy levels after a longer period of time from the diagnosis of GDM. The correlation between the levels of Hcy and carbohydrate disorders or nourishment status in women with pGDM have not been investigated.

## Aim of the study

The aim of the study was to evaluate Hcy levels in women from a central-European population diagnosed with pGDM many years before and to compare these levels with their current carbohydrate metabolism and nourishment status.

## Materials and methods

### Study population

The study group comprised 199 women who had given birth to their children within the last 5–12 years and had been diagnosed with GDM based on an oral glucose tolerance test (OGTT) conducted during pregnancy (pGDM group). The control group included 50 women of comparable age who had given birth during the same period of time and in whom GDM was excluded based on OGTT results during pregnancy (C group). The study had the approval of the Bioethics Committee of the Pomeranian Medical University, and written informed consent was obtained from all participants.

## Methods

All study participants were interviewed to gather information on the number and course of their pregnancies. The physical examination included an assessment of body mass with the calculation of body mass index [BMI (kg/m^2^)] and the measurement of waist and hip circumference with the calculation of waist/hip ratio (WHR). Body composition was measured using a Tanita SC-330S analyzer (Tanita Corporation, Tokyo, Japan). An oral glucose tolerance test (OGTT, 75 g) was performed in all women. The OGTT analyzed the glucose level (enzymatic method, Cormay SA, Warsaw, Poland) and the insulin level (IRMA method, BioSource Europe SA, Nivelles, Belgium) at 0, 60 and 120 min of the test. Baseline glucose and insulin levels were used to calculate the insulin resistance index (HOMA-IR) (the HOMA Calculator—Software v2.2.2) [21]. Glycated hemoglobin (HbA1c) was measured using the HPLC technique (Bio-Rad Laboratories, Munich, Germany). Homocysteine levels were measured with the ELISA technique (Bio-Rad Laboratories, Munich, Germany), and creatinine levels were measured using the kinetic alkaline picrate method (Integra kit, Roche, Rotkreuz, Switzerland). Creatinine clearance was calculated based on the Cocroft-Gault equation. The study included only women with normal plasma creatinine levels (<106 µmol/L).

### Statistical analysis

STATISTICA version 7.1 software (StatSoft Inc., Tulsa, OK, USA) was used for database management and statistical analysis. The Mann–Whitney test and Chi-squared test were used for comparison of continuous and nominal variables, respectively. Correlations between continuous variables in each group were analyzed using Spearman’s rank correlation coefficient (Rs). Multiple linear regression model was used for multivariate analysis adjusted for age. A logarithmic transformation was applied for variables with non-normal distribution. A value of <0.05 was considered to be statistically significant. Our study had 80 % statistical power to detect true differences in mean Hcy concentration between the study and control group equal to 1.6 µmol/L.

## Results

Both groups were tested after approx. 7.8 years of an indexed pregnancy, were of comparable age, had a similar number of pregnancies and births, and had similar anthropometric characteristics and body compositions (Table 1). They also had similar degrees of insulin resistance as reflected by HOMA-IR, lipids and creatinine levels. In women with pGDM, the mean levels of HbA1c, fasting glucose, and glucose between 60 and 120 min after the OGTT were significantly greater than in the controls, but still within the normal range. No difference in the Hcy levels in either group was found (Table 1).

**Table 1:** Characteristics of studied groups

Parameter	C (mean ± SD, n = 50)	pGDM (mean ± SD, n = 199)	*p*
Age (years)	36.8 ± 5.6	38.4 ± 6.6	NS
Number of pregnancies	1.9 ± 2.1	2.4 ± 1.4	NS
Number of deliveries	1.8 ± 2.4	2.1 ± 1.2	NS
Time since pGDM/pregnancy (years)	7.8±1.0	7.4 ± 0.7	NS
Body weight before pregnancy (kg)	61.6 ± 11.1	63.3 ± 13.1	NS
Body weight (kg)	68.7 ± 14.9	67.9 ± 15.2	NS
BMI before pregnancy (kg/m^2^)	21.9 ± 3.9	22.5 ± 4.1	NS
BMI (kg/m^2^)	25.4 ± 5.0	25.5 ± 5.6	NS
WHR	0.84 ± 0.07	0.86 ± 0.09	NS
Adipose tissue mass (kg)	22.6 ± 11.1	21.9 ± 10.2	NS
Lean body mass (kg)	46.1 ± 4.8	45.5 ± 6.1	NS
HbA1c (%)	5.4 ± 0.4	5.6 ± 0.4	<0.01
Glucose 0’ (mmol/L)	4.9 ± 0.6	5.3 ± 0.7	<0.0001
Glucose 60’ (mmol/L)	5.8 ± 1.9	8.3 ± 2.6	<0.0001
Glucose 120’ (mmol/L)	4.8 ± 1.1	6.4 ± 2.2	<0.0001
Insulin 0’ (µIU/mL)	13.7 ± 8.5	13.7 ± 8.7	NS
Insulin 60’ (µIU/mL)	83.8 ± 41.1	106.8 ± 62.6	<0.03
Insulin 120’ (µIU/mL)	47.5 ± 30.6	74.6 ± 58.7	<0.002
HOMA-IR	1.73 ± 1.03	1.76 ± 1.05	NS
Homocysteine (µmol/L)	7.56 ± 3.55	7.63 ± 2.66	NS
Total cholesterol (mmol/L)	4.99 ± 0.88	4.93 ± 1.0	NS
HDL-cholesterol (mmol/L)	1.71 ± 0.47	1.78 ± 0.47	NS
LDL-cholesterol (mmol/L)	2.79 ± 0.8	2.68 ± 0.93	NS
Triglycerides (mmol/L)	1.06 ± 0.97	1.11 ± 0.69	NS
Creatinine (µmol/L)	56.6 ± 9.7	58.3 ± 9.7	NS
Creatinine clearance (mL/min)	132.5 ± 33.2	126.3 ± 32.0	NS

*BMI* body mass index, *C* control group, *HbA1c* glycated hemoglobin, *HOMA-IR* homoeostasis model assessment insulin resistance, *pGDM* women with previous gestational diabetes, *WHR* waist/hip ratio

Based on the result of the current OGTT and in accordance with the WHO guidelines [22], the study population was divided into 4 subgroups: normal glucose tolerance (NGT), impaired fasting glucose (IFG), impaired glucose tolerance (IGT), and diabetes mellitus (DM).

Disorders of carbohydrate metabolism based on the results of the OGTT were more frequent in the pGDM group (43.2 vs 12.0 %, ξ^2^ = 18.7, p < 0.001) (Table 2). Another analysis was based on the current BMI of women categorized as normal body mass (NW), overweight (OW) and obese (OB). In both the study and control groups the percentage of women with normal body mass and those that were overweight and obese was comparable (ξ^2^ = 0.18; NS) (Table 2).

**Table 2:** The frequency of glucose metabolism and nutrition disorders in studied groups

	C (n = 50)	pGDM (n = 199)	p
OGTT result			
NGT n (%)	44 (88.0 %)	113 (56.8 %)	ξ^2^ = 18.7, p < 0.001
IFG n (%)	5 (10.0 %)	40 (20.1 %)	
IGT n (%)	1 (2.0 %)	33 (16.6 %)	
DM n (%)	0 (0.0 %)	13 (6.5 %)	
BMI			
NW n (%)	27 (54.0 %)	114 (57.3 %)	ξ^2^ = 0.18, NS
OW n (%)	14 (28.0 %)	48 (24.1 %)	
OB n (%)	9 (18.0 %)	37 (18.6 %)	

*BMI* body mass index, *C* control group, *DM* diabetes mellitus, *IFG* impaired fasting glucose, *IGT* impaired glucose tolerance, *NGT* normal glucose tolerance, *NW* normal body mass, *OB* obesity, *OGTT* oral glucose tolerance test, *OW* overweight, *pGDM* women with previous gestational diabetes

Homocysteine levels in the pGDM group were estimated in subgroups of different carbohydrate metabolism disorders (IFG, IGT, DM) and were comparable and did not differ from the values found in women with NGT (Table 3). The analysis of the NTG-pGDM (n = 113) and NTG-C (n = 44) subgroups also revealed no differences in the homocysteine levels (7.7 ± 2.5 and 7.6 ± 3.6 µmol/L; respectively, NS). There were significant differences in terms of age, BMI, HOMA-IR, HbA1c, HDL-cholesterol, triglycerides and creatinine clearance levels between women with carbohydrate metabolism disorders and those with normal glucose tolerance (Table 3).

**Table 3:** Homocysteine and lipids’ levels in subgroups categorized according to the current OGTT results in pGDM group

Parameter	NGT-pGDM (mean ± SD, n = 113)	IFG-pGDM (mean ± SD, n = 40)	IGT-pGDM (mean ± SD, n = 33)	DM-pGDM (mean ± SD, n = 13)
Age (years)	37.4 ± 6.5	39.9 ± 6.2*	39.9 ± 6.4*	38.7 ± 8.3
BMI (kg/m^2^)	23.9 ± 4.3	26.6 ± 6.2*,^^^	28.1 ± 6.4**,^^^^	29.6 ± 6.8**,^^^^
HOMA-IR	1.51 ± 0.88	1.70 ± 0.86*,^^^	2.24 ± 1.31**,^^^^	2.62 ± 1.38**,^^^^
HbA1c (%)	5.53 ± 0.34	5.70 ± 0.39*,^^^	5.76 ± 0.38**,^^^^	6.19 ± 0.76**,^^^^
Homocysteine (µmol/L)	7.73 ± 2.53	7.84 ± 3.02	7.24 ± 2.92	7.09 ± 1.88
Total cholesterol (mmol/L)	4.98 ± 1.08	5.03 ± 0.99	5.14 ± 1.03	5.16 ± 0.99
HDL-cholesterol (mmol/L)	1.93 ± 0.47	1.79 ± 0.46	1.59 ± 0.49**,^^^^	1.43 ± 0.28**,^++,^^,#^
LDL-cholesterol (mmol/L)	2.64 ± 1.0	2.74 ± 0.93	2.80 ± 0.78	3.03 ± 0.80
Triglycerides (mmol/L)	0.89 ± 0.39	0.92 ± 0.40	1.61 ± 1.14**,^+,^^,##^	1.51 ± 0.74**,^+,^^^
Creatinine (mmol/L)	57.46 ± 9.72	58.34 ± 9.72	58.34 ± 7.96	57.46 ± 7.07
Creatinine clearance (mL/min)	120.8 ± 28.7	127.7 ± 32.9	134.9 ± 32.5*,^^^^	150.4 ± 43.7*,^^^

*BMI* body mass index, *C* control group, *DM* diabetes mellitus, *HbA1c* glycated hemoglobin, *HOMA-IR* homoeostasis model assessment insulin resistance, *IFG* impaired fasting glucose, *IGT* impaired glucose tolerance, *NGT* normal glucose tolerance, *OGTT* oral glucose tolerance test, *pGDM* women with previous gestational diabetes

*p < 0.05 vs NGT, ** p < 0.01 vs NGT

^+^p < 0.05 vs IFG, ^++^ p < 0.01 vs IFG

^^^p < 0.05 vs NGT (adjusted for age), ^^^^ p < 0.01 vs NGT (adjusted for age)

^#^p < 0.05 vs IFG (adjusted for age), ^##^ p < 0.01 vs IFG (adjusted for age)

There were no statistically significant differences in the homocysteine levels in different BMI subgroups. Women with a greater BMI were significantly older and had higher values of HOMA-IR, LDL-cholesterol, triglycerides and creatinine clearance but had lower HDL-cholesterol (Table 4).

**Table 4:** Homocysteine and lipids’ levels in subgroups categorized according to BMI in pGDM group

Parameter	BMI < 25 (mean ± SD, n = 114, NW)	BMI 25–29.9 (mean ± SD, n = 48, OW)	BMI ≥ 30 (mean ± SD, n = 37, OB)
Age (years)	37.4 ± 6.2	38.9 ± 7.6	40.6 ± 5.9**
BMI (kg/m^2^)	21.6 ± 1.9	27.6 ± 1.2**	34.9 ± 3.9**,^++^
HOMA-IR	1.42 ± 0.73	1.70 ± 1.0**	2.60 ± 1.38**,^++^
HbA1c (%)	5.53 ± 0.35	5.68 ± 0.35*	5.94 ± 0.57**,^+^
Homocysteine (µmol/L)	7.66 ± 2.55	7.23 ± 2.08	8.12 ± 3.58
Total cholesterol (mmol/L)	4.96 ± 1.08	4.96 ± 1.02	5.17 ± 0.87
HDL-cholesterol (mmol/L)	1.97 ± 0.45	1.63 ± 0.41**	1.51 ± 0.45^**^
LDL-cholesterol (mmol/L)	2.57 ± 0.97	2.76 ± 0.94	3.01 ± 0.76^**^
Triglycerides (mmol/L)	0.93 ± 0.44	1.24 ± 0.97**	1.42 ± 0.73**
Creatinine (mmol/L)	57.46 ± 8.84	56.58 ± 8.84	61.0 ± 10.61
Creatinine clearance (mL/min)	109.8 ± 22.3	139.8 ± 28.2**	161.2 ± 30.1**,^++^

*BMI* body mass index, *C* control group, *DM* diabetes mellitus, *HbA1c* glycated hemoglobin, *HOMA* *IR* homoeostasis model assessment insulin resistance, *IFG* impaired fasting glucose, *IGT* impaired glucose tolerance, *NGT* normal glucose tolerance, *NW* normal body mass, *OB* obesity, *OW* overweight, *pGDM* women with previous gestational diabetes

* p < 0.05 vs NW, ** p < 0.01 vs NW

^+^p < 0.05 vs OW, ^++^ p < 0.01 vs OW

No statistically significant correlation was found between homocysteine levels and body mass, BMI, WHR, content of body fat or non-adipose tissue. Moreover, no correlation was found between the homocysteine levels and the metabolic parameters of glucose and insulin levels during the OGTT, HbA1c, HOMA IR index, and parameters of lipid metabolism. A positive correlation was found between Hcy and creatinine levels (*r* = 0.21, p < 0.004) and a negative correlation was found between Hcy and creatinine clearance (*r* = −0.16, p < 0.03). The multi-factor analysis of variance adjusted for age revealed that higher Hcy levels were associated only with higher plasma creatinine levels (β = 0.25, 95 % CI 0.11–0.39, *p* < 0.001).

## Discussion

A history of GDM is associated with an increased risk of type 2 diabetes. In the 10 years after the end of a pregnancy complicated by GDM, 20–70 % of women may have diabetes mellitus [1, 4, 23]. Our study was carried out approximately 8 years following the diagnosis of pGDM revealed carbohydrate metabolism disorders in approximately 43 % of the subjects, but only 6.5 % of the women had diabetes. It can be assumed that the low prevalence of diabetes in the pGDM group could result from the fact that the majority of women (57 %) had normal body mass, whereas 24 % were overweight and approximately 19 % were obese.

It is not clear whether the increased risk of atherosclerosis and cardiovascular complications in women with pGDM results from carbohydrate metabolism disorders developing or worsening over time, or whether it is related to other metabolic disorders. Hcy, which has proatherogenic activity, may be one such risk factor. The relationship between homocysteine levels and insulin resistance and the components of the metabolic syndrome has been demonstrated in a study on a general population [13]. In a prospective study of 170 Korean women with pGDM who were evaluated 4 years following childbirth, 10.8 % were diagnosed with diabetes mellitus and independent risk factors for developing diabetes and displayed elevated Hcy and elevated fasting glucose measured soon after delivery [19]. A study carried out in Iran in women with pGDM evaluated 4 years following childbirth revealed significantly higher levels of Hcy compared with the control group [20]. Homocysteine levels were higher in women with impaired glucose tolerance. However, the findings from studies assessing the relationship between homocysteine levels and carbohydrate metabolism disorders are inconsistent. Nevertheless, most of them do not confirm any correlation [24–26].

Our study did not reveal any significant relationship between homocysteine levels and the severity of carbohydrate metabolism disorders, components of the metabolic syndrome, or current composition of body mass in women with a history of GDM. However, Hcy levels were positively correlated with plasma creatinine levels and negatively correlated with creatinine clearance levels. Another study of subjects at risk for diabetes (family history of DM, overweight or women with pGDM) demonstrated a similar finding [27]. It is known that renal function affects the blood Hcy level [28]. However, it was still surprising that the correlation between Hcy levels and glomerular filtration rate observed in our study was so strong in young women without comorbidities and creatinine levels within the normal range. Significantly higher values of creatinine clearance were found in women with more severe carbohydrate metabolism disorders and in overweight and obese women. The increased glomerular filtration rate (GFR) observed in these groups resulted from greater body mass and perhaps higher glucose levels [29]. The increased GFR observed in these groups may exert a protective effect and prevent the increase in homocysteine levels in the blood. Slowly decreasing GFR with age may lead to an increase in Hcy levels, with subsequent adverse effects on the micro- and macrocirculation in patients with type 2 diabetes [26, 30–32].

The strong points of our study include the large homogeneous population of women with a history of GDM, a precisely selected control group and a wide panel of anthropometric and metabolic tests. The study also has some limitations. It did not consider factors affecting Hcy levels such as levels of vitamin B6, B12, and folic acid, the use of stimulants or diet. Nevertheless, all of the analyzed women had no comorbidities and followed a typical central European diet; these additional parameters were not considered in other, similar studies.

## Conclusions

The study revealed that in women with a history of gestational diabetes, homocysteine is not a marker of increased cardiovascular risk, as its levels are not correlated with current carbohydrate metabolism disorders, overweight status or obesity. Increased GFR was related to more severe disorders of carbohydrate metabolism and greater body mass being risk factors for cardiovascular diseases, it may temporarily protect against an increase in the level of proatherogenic homocysteine.

## Abbreviations

BMI: body mass index
C: control group
DM: diabetes mellitus
GDM: gestational diabetes
GFR: glomerular filtration rate
HOMA-IR: homoeostasis model assessment insulin resistance
Hcy: homocysteine
IFG: impaired fasting glucose
IGT: impaired glucose tolerance
NGT: normal glucose tolerance
NW: normal body mass
OB: obesity
OGTT: oral glucose tolerance test
OW: overweight
pGDM: previous gestational diabetes
WHR: waist/hip ratio
